# ‘If we don't have consent, we need to have beneficence’: Requiring beneficence in nonconsensual neurocorrection

**DOI:** 10.1111/bioe.13043

**Published:** 2022-05-19

**Authors:** Emma Dore‐Horgan

**Affiliations:** ^1^ Uehiro Centre for Practical Ethics University of Oxford Oxford UK

**Keywords:** beneficence, consent, criminal rehabilitation, neurocorrectives, neurolaw, protections against abuse

## Abstract

Neurointerventions—interventions that cause direct physical, chemical or biological effects on the brain—are sometimes administered to criminal offenders for the purpose of reducing their recidivism risk and promoting their rehabilitation more generally. Ethical debate on this practice (henceforth called ‘neurocorrection’) has focused on the issue of consent, with some authors defending a consent requirement in neurocorrection and others rejecting this. In this paper, I align with the view that consent might not always be necessary for permissible neurocorrective use, but introduce a qualification I argue ought to inform our ethical and legal analysis of neurocorrection if we *are* to administer neurocorrectives nonconsensually. I maintain our use of nonconsensual neurocorrection should be constrained by a beneficence requirement—that it should be limited to neurocorrectives that can be expected to benefit those required to undergo them; and my argument is that a beneficence requirement is necessary in order to safeguard against offender abuse. I highlight how we afford a heightened protective role to beneficence in other instances of biomedical intervention where consent is absent or in doubt; and I argue a beneficence requirement is also necessary in the correctional context because alternative candidate protections would provide insufficiently strong safeguards on their own. I then consider whether requiring beneficence in nonconsensual neurocorrection would (a) be incompatible with penal theory, (b) be objectionably paternalistic, or (c) foreclose many fruitful avenues of crime control. I argue in each case that it would not.

## 1 INTRODUCTION

Neurointerventions—interventions that exert direct physical, chemical or biological effects upon the brain^1^—are sometimes deployed by the institutions of criminal justice for the purpose of reducing the recidivism risk posed by offenders or promoting their rehabilitation more generally. Two such ‘neurocorrectives’ are currently in use beyond the research phase in some jurisdictions. In the United Kingdom, for example, courts have the sentencing option of a Drug Rehabilitation Requirement,^2^ wherein substance‐abusing offenders are obliged to submit to opioid substitution therapy in order to curb their addiction and consequently facilitate their rehabilitation. And in some jurisdictions in the United States and Europe, sexual offenders can be required to undergo antilibidinal intervention (ALI) or ‘chemical castration’ either as part of their sentences or as a condition of parole or release.^3^ These examples, however, may be just the beginning. There is reason to believe a much wider range of ‘neurocorrectives’ will become available as neuroscientific and pharmaceutical knowledge advances—for example, pharmaceutical and/or brain stimulation interventions to improve impulse control, enhance empathy or attenuate aggression.^4^ Unsurprisingly, questions about whether and how we should deploy these interventions have been the subject of much scholarly debate.

This ethical debate has given rise to two broad positions with respect to the use of *nonconsensual* neurocorrection—that is neurocorrection administered without the valid (or free and informed) consent of offenders. This would be where, for instance, neurocorrection is mandated as part of an offender's sentence, or where coercive pressure is placed on offenders to submit to neurocorrection by making it a condition of their parole or early release.^5^ On one side, some authors argue against nonconsensual neurocorrection on the grounds that it involves an unjustifiable infringement of offender rights (specifically the rights to bodily and/or mental integrity),^6^ and/or is objectionably disrespectful of offenders as rational and autonomous agents.^7^ On the other side, it is argued the State sometimes has strong public protection reasons for administering neurocorrectives even when consent is absent, and the aforementioned rights and respect‐based objections do not count against the use of nonconsensual neurocorrection across the board. Some authors argue, for example, that in committing crime, offenders render themselves liable to some sort of intrusive and/or defensive action on the part of the State, and that this could include liability to neurocorrection when such intervention is a necessary and proportionate response for the protection of the public.^8^ Others argue that nonconsensual neurocorrection is not necessarily impermissibly disrespectful.^9^

In this paper, I align with the latter camp in holding that a strong moral case can be made for the permissibility of nonconsensual neurocorrection when offenders would pose a serious risk to the public upon societal re‐entry. However, I introduce a qualification I maintain ought to inform our ethical and legal analysis of nonconsensual neurocorrection. I argue that in deploying neurocorrectives nonconsensually we should be constrained by a beneficence requirement in order to safeguard against offender abuse. In other words, our use of nonconsensual neurocorrection should, for safeguarding reasons, be limited to neurocorrectives that can be expected to benefit those required to undergo them.

This precise line of argument has not been developed in depth before. Some authors have maintained neurocorrectives need *not* be in an offender's ‘best interests’ for penal‐theoretic reasons. Petersen has claimed that consequentialist and retributivist theories of punishment ‘allow[] for situations where it would be morally right to offer a [neurocorrective] that is not in the best interests of the offender’.^10^Vallentyne maintains neurocorrectives would not wrong their targets if ‘the harm impose[d] on the agent is proportionate’ and ‘necessary…for achieving certain moral goals’ such as the reduction of future harms to others.^11^ And Pugh and Douglas likewise indicate that ‘culpably pos[ing] a significant risk to the life of [others] …may be enough to justify imposing … harmful preventative interventions’ on offenders.^12^Other authors, in contrast, have maintained that morally acceptable neurocorrection must be ‘in the best interests of the offender’,^13^ ‘substantially improving his or her overall prospects as compared with [traditional] punishment’.^14^ But these authors *also* stipulate the need for a consent requirement in neurocorrection and their arguments are rights‐based—they claim that consent and beneficence are *both* necessary if we are to avoid violating offenders' human rights.

My argument here differs from both these stances in that it defends a beneficence requirement in nonconsensual neurocorrection on purely instrumental grounds. I am not contending offenders have a *right* that neurocorrectives be beneficial, nor am I claiming that rights‐based considerations necessarily preclude their nonconsensual use. Instead, I argue against the use of even proportionately harmful nonconsensual neurocorrection on the grounds that, *absent* beneficence in nonconsensual treatment, we have insufficient protections against abuse. I believe this argument is important and timely because there is currently legal scope (in some jurisdictions) to administer hormonal treatments nonconsensually to sexual offenders for whom such treatment would *not* be beneficial.^15^ This situation should, I contend, be remedied before the range of neurocorrectives at our disposal broadens.

My discussion is organized as follows. In Section 2, I outline my argument for a beneficence requirement. I highlight how we afford a heightened protective role to beneficence in other instances of biomedical intervention where consent is absent or in doubt. And I argue a beneficence requirement is *also* necessary in the correctional context if we are to ensure sufficiently strong protections against abuse. Section 3 anticipates and addresses the following potential objections: that requiring beneficence in nonconsensual neurocorrection would (a) be incompatible with penal theory, (b) be objectionably paternalistic, and (c) foreclose potentially fruitful avenues of crime control. Section 4 concludes.

Some clarificatory comments before we proceed. This paper assumes throughout that the purpose of neurocorrectives is *primarily* rehabilitative. I take it our principal reason for using neurointerventions in criminal justice is to facilitate dispositional changes in offenders in order to reduce crime and/or its social costs, and *not* to find new and additional ways of punishing these individuals. I assume further that the goal of crime reduction is primarily pursued for the benefit of society, while also acknowledging that rehabilitation through neurocorrection might sometimes be pursued solely for the benefit of offenders themselves (such as when, e.g. opioid‐substitution therapy is made available to an offender who poses no great risk of future recidivism but for whom recovery from addiction would be beneficial). My concern in this paper, however, is with the former kinds of situations: to argue for a beneficence requirement in situations where crime prevention is the primary ground for intervention, and where neurocorrection is pursued in the absence of valid consent.

I additionally assume that when it comes to *nonconsensual* neurocorrection, this intervention can also constitute punishment or part‐punishment because of the liberty and autonomy restrictions necessarily intended and involved. And I argue briefly (in Section 3) that these punishments or part‐punishments would be compatible with penal theory even if subject to a beneficence requirement.

I concede there may be other instrumental reasons for requiring beneficence in neurocorrection (aside from safeguarding) that I do not rely upon. Beneficial neurocorrectives might, for example, be *more effective* in facilitating rehabilitation and the reduction of recidivism than harmful ones. Nonbeneficial neurocorrection might also undermine both offenders' and the wider public's trust in healthcare and, in so doing, precipitate poorer healthcare outcomes. But I do not utilize these points to support my argument for a beneficence requirement because we lack the necessary empirical evidence. It is plausible that *some* nonbeneficial interventions might render offenders less disposed to engage in crime even if it is also intuitively plausible that offenders with an improved level of well‐being could be less likely to recidivate. And while it is credible that nonbeneficial neurocorrection would at least erode *offenders'* trust in healthcare professionals, the empirical fact of the matter in the case of both offenders and the wider public would still depend on whether the physicians involved are seen to relate to those who carry out standard clinical procedures.^16^

In speaking of nonconsensual yet beneficial neurocorrectives, moreover, I am rejecting the thought that a state of affairs can only ever be good for a person if they desire and validly consent to it. I assume a person can be harmed through nonconsensual treatment yet still receive overall benefit depending on the specifics of the treatment and situation. I am thus rejecting some desire‐based accounts of well‐being which hold that well‐being consists solely in the satisfaction of one's informed desires.

Finally, I do not consider a requirement for beneficence in nonconsensual neurocorrection to be equivalent to a requirement of ‘therapeutic necessity’.^17^ Certainly, treatments that are necessary for managing recognized diseases or disorders typically benefit their recipients.^18^ But not all candidate neurocorrectives target recognized diseases or disorders and it would be a mistake to think that this precludes the possibility of their being beneficial for a given recipient. We can readily agree, for example, that treatment that serves to attenuate a person's impulsively aggressive behaviour (and that does not otherwise render them depressed, apathetic, debilitated or incompetent) plausibly benefits that individual insofar as it enhances their control over their emotions and behaviour in response to provocation. And we can agree upon this even if that individual does not meet the criteria for diagnosis of a recognized disorder of aggression.^19^ Requiring beneficence in nonconsensual neurocorrective use is thus not the same as requiring that neurocorrection be limited to the treatment of acknowledged diseases/disorders. Though, of course, in many instances, beneficial neurocorrection could be coextensive with such treatment.

## 2 PROTECTING AGAINST OFFENDER ABUSE

It is uncontroversial to state that neurocorrectives, like all biomedical interventions, require protections against abuse. These interventions impact upon the most intimate spheres of human life (i.e. one's bodily and mental states); they are capable of exerting profound changes in those they target; and their targets are in a position of supreme vulnerability both because of the significance of the intervention's outcome for them, and because of the imbalance of power and control over the outcome that characterizes the doctor–patient relationship. The history of biomedicine, moreover, is replete with instances in which this capacity and power has been used abusively. There are the numerous atrocities of Nazi medicine,^20^ the infamous Tuskegee syphilis study (where participants unknowingly received placebos rather than treatment for syphilis),^21^ and the notorious use of prefrontal lobotomies to treat socially undesirable behaviour,^22^ to offer but a few examples.

This dark history, together with the fact of patient vulnerability, explains (at least on one narrative) why biomedicine has undergone a medical ethics revolution, investing itself with several legal and codified safeguards against abusive practice.^23^ The work of practitioners and researchers is nowadays expected to be governed by four canonical principles, namely respect for patient autonomy, beneficence, nonmaleficence and justice.^24^ The law, moreover, reflects these principles in making medical intervention in competent adults without informed consent a criminal offence (respect for autonomy); in deeming physicians liable to negligence claims (civil or criminal) if they breach their duty of care to their patients (beneficence and nonmaleficence); and in requiring that practitioners do not discriminate against patients in the delivery of care (justice).^25^

The requirement for valid consent to medical investigations and treatment is arguably the key pillar of ethical biomedicine in contemporary practice. However, there are some very limited instances in which a consent requirement does not (and sometimes cannot) assume an overarching guiding role. A requirement for informed consent is not invoked in the case of individuals who lack the capacity to give informed consent for various reasons.^26^ There are also some limited (controversial) instances where competent adults can be required to undergo clinical assessment or intervention in nonvoluntary circumstances, though in these instances they are still required to be fully informed.

Mental health law in several jurisdictions, for example, allows for the involuntary detention and treatment of mentally ill patients who pose a threat to themselves and/or others.^27^ Public health law also sometimes permits that patients suffering from communicable diseases be detained against their will (and sometimes, controversially, be involuntarily treated).^28^ And criminal law sometimes allows for the delivery of psychotherapeutic interventions in the absence of offenders' free or voluntary consent. Domestic violence perpetrators can be ordered by the courts to attend ‘batterer interventions programs’ (BIPs) involving cognitive behavioural therapy or counselling.^29^ Sexual offenders can likewise be required to undergo various psychosocial therapies as part of their sentences or as a condition of parole or early release.^30^ The law, of course, does not allow that offenders be *physically forced* to submit to rehabilitative interventions. *Compelled* rehabilitation in this absolute sense is thus almost impossible under existing legal arrangements. There can, however, be negative consequences for offenders who do not participate or who fail to accept the terms of their parole or probation. This means it is not always required that offenders *freely* consent to intervention, even if a right to refuse treatment is normally upheld.

I maintain that when a requirement for valid consent to medical intervention is absent, the need to bolster alternative protections is paramount regardless of whether the intervention is for standard clinical, public health or correctional purposes. And if we are to include neurocorrection among the limited contexts in which involuntary medical intervention is (controversially) permitted, we must ensure that adequate and well‐defined alternative protections govern its usage. I argue a beneficence requirement is an apt and necessary protection to adopt in this context—first because requiring beneficence would be consistent with our practices in other permitted instances of involuntary medical intervention, and second because alternative candidate protections are inadequate on their own. Let me elaborate.

First, beneficence is typically afforded a heightened protective role in other instances where consent to medical intervention is absent or in doubt. This gives us at least presumptive reason to favour requiring beneficence in nonconsensual neurocorrection. In situations where someone is *incapable* of validly consenting to healthcare interventions, for example, the UK's mental capacity legislation requires that any action undertaken must ‘benefit the adult’ personally^31^ or be in their ‘best interests’.^32^ And in rare clinical research instances where the *validity* of consent is in doubt (as might be the case, some argue, when *prisoners* partake in clinical trials),^33^ various legal instruments and ethical guidelines emphasize the centrality of a beneficence requirement in facilitating ethical practice. The U.S. Code of Federal Regulations stipulates that research involving prisoners ‘needs to have the intent and reasonable probability of improving the health or well‐being’ of participants;^34^ and the *International ethical guidelines for biomedical research involving human subjects* states that when the validity of consent is dubious, research ought to afford ‘direct benefit’ to participants.^35^

It might be pointed out here that the above examples are selective—that there are occasions of involuntary intervention in competent adults where beneficence is not afforded as pivotal a role. When individuals pose a threat to others, for example either through their direct actions or when they harbour a dangerous infectious disease, efforts to assess and contain the threat are not required to benefit the person targeted. Instead, the goal is public protection, and protection for the individual is provided—not through a beneficence requirement—but by requiring that intervention be the ‘least restrictive’ option for assuaging the threat posed, that efforts to ‘maximise [the individual's] independence’ are made, and that patients are ‘treated with respect and dignity’.^36^ Given that neurocorrection is more akin to these kinds of interventions in virtue of its public protection aims, it might be claimed a requirement to benefit the individual targeted is inessential in this context too.

However, such a line of thought mischaracterizes current practice when it comes to the medical management of *chronic* threats to third parties or public health and this is of relevance for neurocorrection. Certainly, intervention in emergency or acutely threatening situations is not required to benefit the threatening party. But when it comes to managing chronic or continued threats to others, a beneficence requirement still plays a decisive role in current mental health and public health regimes. To give illustrative examples, England's accompanying Code of Practice to MHA 1983 repeatedly emphasizes the need for ‘clear benefits for patients’ in decisions regarding ongoing care and management—with community treatment orders only expected to be used ‘when there is reasonable evidence to suggest … there will be benefits to the individual’.^37^ Public health legislation similarly only allows for mandatory vaccination against (or supervised treatment of) grave infectious disease threats when the safety profile of the proposed intervention is high and clear benefits are also to be obtained for the individual who submits to it.^38^ My point here is that, given neurocorrection is similar to the above contexts insofar as it aims to manage chronic threats biomedically, requiring beneficence in its use would be consistent with existing protective practices in these other regimes. I thus affirm we have at least presumptive reason to favour a beneficence requirement in nonconsensual neurocorrection.

I imagine the reader at this point will be contemplating the obvious disanalogies between neurocorrection and healthcare, and querying whether safeguarding in the *correctional* context warrants a beneficence requirement. It might be pointed out that if neurocorrection is part of a sentence or a term of parole then it is inescapably tied up with penal theoretic considerations; and safeguarding against abuse when an intervention is serving at least *some* punitive function is not usually thought to necessitate beneficence. We protect against abuse elsewhere in the correctional context, after all, by requiring that any harms incurred are neither disproportionate to the offender's crime nor to the societal threat they pose.^39^ We also often demand these harms be no more severe or restrictive than alternative, effective means of reducing the offender's recidivism risk. Why not then rely on a proportionality requirement and/or a ‘least harmful’ alternative requirement in nonconsensual neurocorrection instead of insisting that neurocorrectives produce benefit for their targets?

I contend the above constraints would not provide *strong enough* protections against the abusive use of neurocorrectives notwithstanding the importance afforded to them in corrections more generally.

Consider first proportionality, and reflect upon how many plausible targets for nonconsensual neurocorrection come from particularly reviled and feared subpopulations for whom the danger of *overestimating* recidivism risk and liability to defensive harm is ever‐present. I say these subpopulations are the most plausible targets because, first, proponents of nonconsensual neurocorrection have only defended their use for serious crimes;^40^ and second, the moral case for imposing neurocorrectives on offenders becomes, I believe, substantially weaker in cases where offenders have *not* committed very serious offences and/or do not pose a significant threat to the public if not rehabilitated.^41^ In primarily targeting these reviled and feared subgroups through neurocorrection, then, we are in very real danger of overestimating liability and risk both because these groups are comprised of individuals for whom we (the public and sentencers) have little sympathy, and because *underestimating* these individuals' level of risk would have significant human costs. Clinical assessments of reoffending risks in these populations have been shown to be particularly cautious, with mental health professionals and judges tending to *overpredict* future violence (both nonsexual and sexual).^42^ And while the predictive accuracy for risk is reportedly much improved when clinical and actuarial methods of risk assessment are utilized in tandem (and by the latter, I mean the use of algorithmic risk assessment tools),^43^ mere proportionality considerations might still not prevent our imposing more harm through neurocorrection than is necessary to reduce recidivism risk. Consequently, I contend a mere proportionality requirement would provide insufficient protections against offender abuse.^44^

What then, about a ‘least harmful alternative’ requirement? Would requiring that neurocorrection be the least harmful (effective) means of reducing an offender's recidivism risk afford sufficient protections against abuse? Again, I argue no. Imagine, for instance, that an impulsively violent offender's risk could be reduced by a pharmaceutical that suppressed their violent impulses but which also rendered them apathetic. Suppose further that this is the only effective means of protecting the public from the offender's violent outbursts aside from continued incarceration—that conventional rehabilitative measures such as anger management training or cognitive behavioural therapy would be (or have proven to be) ineffective on their own. In this scenario, nonconsensual neurocorrection might be less harmful than the carceral alternative. It would restore the offender's freedom of movement, better facilitate the preservation of friendship and family ties, and release the offender from the grave physical and mental health threats that prevailing prison conditions often create (though it would still be expected to be at least mildly harmful overall).^45^ Yet, I dispute that favourable comparisons with prevailing practice are anyway helpful in constructing adequate safeguards. This is because I hold, along with others, that current carceral practices are often unjustifiably harmful anyway. Prisoners are frequent victims of violent and/or sexual assault, often serve their sentences in overcrowded living environments, and regularly do not have their mental health issues and needs adequately accommodated for within the prison environs.^46^ To rely on the requirement that neurocorrection be *better* than imprisonment does little to safeguard against its abusive deployment when prevailing practices themselves allow for abuse. And while it might be more humane to allow offenders to undergo neurocorrection rather than long(er) imprisonment in a given instance, in the absence of free and voluntary consent to the former we need stronger protections than that which a mere comparison with the carceral alternative affords. A ‘least harmful alternative’ requirement thus cannot adequately protect against abuse in neurocorrection if the alternatives involve incarceration as it is currently practiced.

The reader might still be unconvinced that the inadequacies of the abovementioned protective measures entail a beneficence requirement is necessary. They might respond that a requirement for mere *nonmaleficence* would suffice. Why, after all, would a neurocorrective have to produce benefits for its recipient when merely requiring that it ‘do no harm’ would also guard against its being used abusively? A beneficence requirement is, in the end, just overkill, so the response might go.

I agree that a nonmaleficence requirement in neurocorrection would offer protection against offender abuse. However, I contend a nonmaleficence requirement in nonconsensual neurocorrection amounts to the same as a beneficence one—that is, it also requires that intervention produce benefits for its target. This is because *nonconsensual* neurocorrection in‐and‐of‐itself falls foul of nonmaleficence. Interfering in the affairs of another without their valid consent is itself a *harm*. Interfering with their body and/or mental states nonconsensually, moreover, is a particularly significant harm and one that is likely to be experienced as very intrusive and unpleasant.^47^ As such, nonconsensual neurocorrection does not satisfy a requirement for nonmaleficence unless the effects of the intervention are sufficiently beneficial as to counteract the harm of nonconsensual treatment. And given that it is improbable that the harm of nonconsensual treatment and the beneficial effects of intervention will map onto each other *exactly*, *ensuring* that no harm is done requires that intervention produce net benefit. Nonmaleficence, in this context, therefore requires beneficence.

The reader might instead object, however, to the need to focus on benefits and harms at all when it comes to protecting against abuse in neurocorrection. They might suggest a requirement to *respect human dignity* would be an appropriate alternative protection and one to be preferred over an approach that involves making substantive judgements about what would be beneficial for others.

I agree that a requirement to respect human dignity should feature prominently in our regulations surrounding neurocorrection. However, I dispute that dignity‐based considerations provide sufficient protections in isolation if we are already administering neurocorrectives nonconsensually. Consider for a moment what respecting human dignity might involve. The commonly voiced thought is that respecting dignity means treating people in a way that befits their status as rational and autonomous agents. For some, this entails respecting their autonomous *choices*; and on such an understanding, neurocorrection in the absence of valid consent necessarily offends against human dignity. For others (including myself), there is still some scope to remain respectful of human dignity even when intervening in another person's affairs against their will. We can still respect offenders' rational and autonomous agency by working to protect and promote their capacity for independence and rational self‐determination in matters apart from the mandatory or conditional terms of their sentences. We can also involve them in discussions pertaining to their neurocorrective treatment and take account of their views regarding some of the specifics of treatment (e.g. their views on which pharmaceutical to use when more than one option is available).^48^ And we can refrain from deploying particularly *invasive* interventions nonconsensually (e.g. psychosurgery or direct brain stimulation), thus avoiding those methods that—due to their substantial infringement upon offenders' bodily integrity—would be invariably disrespectful in the absence of valid consent.

Yet, I still maintain we also need a requirement that references the harms and benefits of intervention if we are to have sufficient protections against abuse. Abuse, after all, is defined as behaviour that is unjustifiably *harmful* to another; and guarding against such behaviour requires that we mitigate the ease with which gratuitous harming can take place. It is not clear to me that a requirement to respect human dignity can achieve this goal alone—that it can provide adequate defences against abuse in isolation from a requirement that references the expected benefits and harms for the individual targeted. We could arguably remain respectful of human dignity when administering noninvasive neurocorrectives nonconsensually if we take appropriate steps to protect offenders' rational and autonomous agency. But this still does not preclude our imposing an *unjustifiably harmful* intervention, given that dignity‐based considerations do not directly reference harms and benefits at all. Avoiding unjustified harming through neurocorrection thus requires that we *also* address what level of harm to offenders we can safely permit. And given the abovementioned real‐world difficulties in relying on an assessment of proportionate harm or in requiring that the ‘least harmful’ course of action be followed, I still contend a beneficence requirement is necessary to forestall the risk of abuse through neurocorrection.

A final difficulty that might be voiced at this juncture concerns the usefulness and adequacy of a beneficence requirement in the absence of a clear account of benefit or ‘best interests’. The worry here is that, in the absence of clear guidance on what constitutes a benefit, a legal and codified requirement for beneficence will not adequately protect against abuse.^49^ I agree. I accept that that we need a clear account of benefit if a beneficence requirement is to do the work I am expecting of it. However, I do not think this presents a problem for my argument as I believe such an account can be provided. And while I lack the space to develop such an account in this paper, a promising starting point would be to look at *other* spheres that are at least partially concerned with welfare promotion; to examine the substantive judgements that these spheres have made about how to improve people's well‐being; and to extrapolate the guidance proffered to the neurocorrective context accordingly. We thus might, for example, look to standard medical practice and public policy approaches to health and education, identify the kinds of things these spheres take to constitute a benefit to individuals, and then assess which of these benefits are also amenable to enhancement through neurocorrection in order produce a clear account with which to work.^50^ This groundwork is a necessary prerequisite if a requirement for beneficence is to serve as an effective safeguard. But once such an account is in place I maintain a beneficence requirement will afford the required protections.

## 3 SOME CHALLENGES CONSIDERED

So far, so good. I have argued, in the preceding section, that nonconsensual neurocorrection should be constrained by a beneficence requirement in order to adequately protect against offender abuse. Now, let us look to objections this argument might invite.

I anticipate three principal objections. The first protests that beneficial neurocorrection, if administered as part of a sentence or as a condition of parole, would be incompatible with theories of punishment. The second maintains that striving to benefit offenders through neurocorrection without their consent would be objectionably paternalistic. The third worries that limiting the nonconsensual use of neurocorrectives to interventions that would be beneficial for offenders would foreclose potentially fruitful avenues of crime control. I shall examine each of these objections in turn.

Looking first to the objection that beneficial, nonconsensual neurocorrection would be incompatible with offender punishment, let me reiterate that I consider the primary purpose of neurocorrection to be rehabilitation, not punishment, but accept that when administered as part of a sentence or a condition of parole (and hence when involving some liberty/autonomy restriction) neurocorrection also serves some punitive purpose. The mere fact that such punishment can be expected to benefit its recipient, however, is not penologically problematic so long as the overall punishment dispensed remains proportionate and capable of furthering punishment's goals.^51^ Additional punishment imposed alongside or prior to beneficial, nonconsensual neurocorrection, after all, could render the overall burdens meted out to be proportionate to the gravity of the offence, and sufficient for deterring crime and communicating censure. And even if the neurocorrective itself is to serve as the principle (or sole) sanction, this would I contend be consistent with penal theory so long as the harmful aspects of intervention (i.e. its nonconsensual administration and any harmful effects) are sufficient for advancing punishment's goals of retribution, deterrence or denunciation.

Could proponents of canonical theories of punishment be content with merely pro tanto harmful punishment such as this? That is, could they be satisfied with punitive measures that elicit some harms but also produce benefits that might ultimately prevail over these harmful features? I think so, though I cannot defend this claim thoroughly here.^52^ Let me suggest, however, that even the retributivist might be satisfied with mere pro tanto harm so long as this harm remains proportionate to the offence and precedes the arrival of any benefits to offenders. Retributivists, after all, do not typically take temporally distant punishment outcomes into account when discussing issues of proportionality. They thus have ‘very little to say’ about what follows *after* punishment has been served.^53^

What about the worry that nonconsensual, beneficial neurocorrection would be objectionably paternalistic? Admittedly, this concern might strike some as odd. Worries about paternalism are unlikely to be at the forefront of one's mind when one contemplates nonconsensual neurocorrection, given that these interventions can be intended as punishment and typically will (as is the case with medical interventions usually) be accompanied by some undesirable, unpleasant or inconvenient side‐effects.^54^ Yet, a critic might insist that nonconsensual yet beneficial neurocorrectives are vulnerable to the charge of paternalism because they (a) aim to further the offender's good irrespective of their own will; and (b) their deployment is motivated by the negative judgement that offenders lack the ability to (and hence will not) pursue their own good through beneficial neurocorrection unless the latter is mandated or incentivized.^55^

In response, let me first state that it is not obvious that nonconsensual, beneficial neurocorrectives necessarily express negative judgements about offenders' prudential abilities. As already indicated, the principal reason for deploying neurocorrectives nonconsensually might be to ensure that that an offender's risk of recidivism is reduced; and as such, little thought might be given to whether the offender would freely choose intervention if it were not mandated or made a condition of parole. Nevertheless, let us assume for the sake of argument that *some* instances of nonconsensual neurocorrection are motivated by this kind of negative judgement and that *all* necessarily intend to further offenders' own good irrespective of their own will. I still do not think that nonconsensual, beneficial neurocorrection is objectionable by virtue of its paternalism so long as the primary purpose of intervention is to prevent harm to *others*. Here, I am allowing (in line with some views) that nonconsensual intervention meets the criteria for paternalism if the good of the recipient is but one reason for acting.^56^ But I dispute that these mildly paternalistic acts are *objectionably* paternalistic if the key reason for intervention is the prevention of other‐directed harms. In the presence of this primary goal, I believe any additional paternalistic intent need not enter into our calculations when it comes to assessments of permissibility. However, when benevolence toward the offender is the sole or primary motivator for acting, I concede there is reason to object to the paternalism involved and hold that further details would be needed to make judgements about overall permissibility in these cases.

Turning finally to the concern about foreclosing potentially effective avenues of crime control, I concede this is something we have to accept, but dispute that a beneficence requirement would *unduly* circumscribe the range of neurocorrectives that could permissibly be deployed nonconsensually. A beneficence requirement would mean we would be prohibited, at least in typical cases, from utilizing neurocorrectives that merely induce apathy in offenders, for example. Yet, this does not limit the prospects for utilizing neurocorrection as a public protection tool excessively. There are many candidate neurocorrectives that would plausibly benefit *some* subpopulations of offenders. Some offenders' lives might go significantly better for them if their impulse control could be improved through neurocorrection. Others' well‐being might increase if neurocorrection could enhance their ability to establish and maintain close connections with others (if, e.g. aggressive tendencies or empathy deficits have hampered this). Requiring beneficence would certainly restrict the populations to whom various neurocorrectives could be administered nonconsensually. But it would not preclude their use entirely, and such restriction is, I believe, a price worth paying if we are to have sufficient safeguards against abuse.

## 4 CONCLUSION

To sum up, this paper has made a case for requiring beneficence in nonconsensual neurocorrection. I first highlighted how a beneficence requirement is afforded a heightened protective role in other situations where consent to biomedical intervention is absent or in doubt. I then argued that a beneficence requirement is necessary to safeguard against abuse in nonconsensual neurocorrection because it affords stronger protection than alternative requirements would in its absence. I finally addressed three potential objections to my line of argument.

I concede we would have greater protections against abuse if neurocorrectives were also subject to a consent requirement. However, I think there are *some* circumstances in which tolerating some risk of misuse would be preferable to avoiding neurocorrection altogether. In the case of those who have committed the most serious of offences, requiring neurocorrection prior to societal re‐integration would be preferable to risking release *sans* intervention, despite the risk of misuse. My argument here is that if we *are* to risk misuse by relinquishing a requirement for consent, we must construct adequate alternative protections. And a beneficence requirement is one necessary foundation for such a structure.

## CONFLICT OF INTEREST

The author declares no conflict of interest.

## Footnotes

1 See Douglas, T., & Birks, D. (2018). Introduction. In D. Birks & T. Douglas (Eds.), *Treatment for crime* (pp. 1–11). Oxford University Press for this definition.

2 Criminal Justice Act 2003, s 209.

3 Tilemann, H. (2016). *Review of laws providing for chemical castration in criminal justice*. Institute of Criminal Justice Reform. https://icjr.or.id/wp-content/uploads/2016/06/paper-ICJR_ResearchNote_CC_CM.pdf; Scott, C. L., & Holmberg, T. (2003). Castration of sex offenders: prisoners' rights versus public safety. *Journal of American Academy of Psychiatry and Law, 31*(4), 502–509.

4 Chew, C., Douglas, T., & Faber, N. S. (2018). Biological interventions for crime prevention. In D. Birks & T. Douglas (Eds.), *Treatment for crime* (pp. 11–43). Oxford University Press.

5 There is disagreement in the literature as to whether neurocorrection imposed as a condition of parole or early release is *inherently* coercive and hence nonconsensual. For the sake of argument in this paper, I assume *some* such instances would be.

6 Shaw, E. (2019). The right to bodily integrity and the rehabilitation of offenders through medical interventions: A reply to Thomas Douglas. *Neuroethics, 12*(1), 97–106; Bublitz, J. C. (2018). ‘The Soul is the Prison of the Body’: Mandatory moral enhancement, punishment, and rights against neurorehabilitation. In D. Birks & T. Douglas (Eds.), *Treatment for crime* (pp. 289–320). Oxford University Press.

7 Shaw, E. (2018). Against the mandatory use of neurointerventions in criminal sentencing. In D. Birks & T. Douglas (Eds.), *Treatment for crime* (pp. 321–337). Oxford University Press.

8 McMahan, J. (2018). Moral liability to ‘crime‐preventing neurointervention’. In D. Birks & T. Douglas (Eds.), *Treatment for crime* (pp. 117–123). Oxford University Press; Douglas, T. (2014). Criminal rehabilitation through medical intervention: Moral liability and the right to bodily integrity. *The Journal of Ethics, 18*(2), 101–122.

9 De Marco, G., & Douglas, T. (2021). The expressivist objection to nonconsensual neurocorrectives. *Criminal Law and Philosophy*; Holmen, S. J. (2020). Respect, punishment and mandatory neurointerventions. *Neuroethics*. *14*(2), 167–176.

10 Petersen, T. S. (2018). Should neurotechnological treatments offered to offenders always be in their best interests? *Journal of Medical Ethics*, *44*(1), 32–36, p. 35.

11 Vallentyne, P. (2018). Neurointerventions, self‐ownership and enforcement rights. In D. Birks & T. Douglas (Eds.), *Treatment for crime* (pp. 124–140). Oxford University Press, p. 131.

12 Pugh, J., & Douglas, T. (2016). Justifications for non‐consensual medical intervention: From infectious disease control to criminal rehabilitation. *Criminal Justice Ethics, 35*(3), 205–229, p. 221.

13 Focquaert, F. (2014). Mandatory neurotechnological treatment: Ethical issues. *Theoretical Medicine and Bioethics*, 35(1), 59–72, p. 67.

14 Rosati, C. S. (1994). A study of internal punishment. *Wisconsin Law Review, 1994*(1), 123–170, p. 147.

15 The consensus is that paraphiliacs are the only subpopulation of sexual offenders for whom ALIs might be beneficial, see Fitzgerald, E. (1990). Chemical castration: MPA treatment of the sexual offender. *American Journal of Criminal Law, 18*(1), 1. Yet, some ALI statutes either fail to differentiate between paraphiliac and nonparaphiliac sex offenders or do not require that offenders be screened for suitability to undergo ALIs by a physician. See, for example, Cal. Penal Code § 645 (2001) and Fla. Stat. § 794.0235 (1997).

16 Ryberg, J. (2019). *Neurointerventions, crime and punishment: Ethical considerations*. Oxford University Press, p. 157.

17 See Forsberg, L. (2021). Anti‐libidinal interventions and human rights. *Human Rights Law Review, 21*(2), 384–408, p. 394, for a discussion of the therapeutic necessity requirement in the case law of the European Court of Human Rights.

18 I say ‘typically’ because it is not clear that treatment that combats a particular disorder but that nonetheless has a plethora of unpleasant and/or debilitating side‐effects is *always* beneficial for its recipient.

19 Such as intermittent explosive disorder, see Americal Psychiatric Association. (2013). *Diagnostic and statistical manual of mental disorders: DSM‐5* (5th ed.). American Psychiatric Publishing.

20 Cohen, N. (1990). Medical experiments. In I. Gutman (Ed.), *Encyclopedia of the Holocaust* (pp. 957–966). Macmillan Library Reference USA.

21 Reverby, S. (2009). *Examining Tuskegee: The infamous syphilis study and its legacy*. University of North Carolina Press.

22 Snow, H. B. (1949). A review of 27 prefrontal lobotomy patients. *Psychiatric Quarterly, 23*(1), 26–34.

23 Schicktanz, S., Michl, S., & Stoff, H. (2021). Bioethics and the argumentative legacy of atrocities in medical history: Reflections on a complex relationship. *Bioethics, 35*(6), 499–507.

24 Beauchamp, T. L., & Childress, J. F. (2013). *Principles of biomedical ethics* (7th ed.). Oxford University Press.

25 Jackson, E. (2019). *Medical law: Text, cases, and materials* (5th ed.). Oxford University Press.

26 See, for example, Mental Capacity Act (MCA) 2005, ss 2 and 3.

27 For example, Mental Health Act (MHA) 1983 (as amended by Mental Health Act 2007).

28 For example, Public Health (Control of Disease) Act 1984, ss 37 and 38.

29 Wilson, D. B., Feder, L., & Olaghere, A. (2021). Court‐mandated interventions for individuals convicted of domestic violence: An updated Campbell systematic review. *Campbell Systematic Reviews, 17*(1), e1151.

30 Clayton, S. L. (2002). Sex offenders. *Corrections Compendium*, *27*(7), 8.

31 Adults with Incapacity (Scotland) Act 2000, s 1(2).

32 MCA 2005, s 1(5).

33 Due to the coercive pressure that might be introduced within the carceral context.

34 45C.F.R. *§ 46.306*.

35 Council for International Organizations of Medical Sciences (CIOMS). (2002). *International ethical guidelines for biomedical research involving human subjects*. https://media.tghn.org/medialibrary/2011/04/CIOMS_International_Ethical_Guidelines_for_Biomedical_Research_Involving_Human_Subjects.pdf

36 These requirements are found in England's Code of Practice for the MHA 1983 but they are representative of many jurisdictions. See Great Britain. Department of Health. (1993). *Code of practice: Mental Health Act 1983*. p. 22. https://www.gov.uk/government/publications/code-of-practice-mental-health-act-1983

37 Ibid: 73, 330.

38 For instance, most vaccine mandates apply to childhood immunization where vaccination is clearly in the child's best interests and well established as safe, see Savulescu, J. (2021). Good reasons to vaccinate: Mandatory or payment for risk? *Journal of Medical Ethics, 47*, 78–85.

39 See U. S. Const. amend. VIII; and Bagaric, M. (2000). Proportionality in sentencing: Its justification, meaning and role. *Current Issues in Criminal Justice, 12*(2), 143–165.

40 Ryberg, op. cit. note 16; Douglas, op. cit. note 8.

41 In these minor cases, the proportionality requirement would likely be violated in any case if neurocorrectives were imposed.

42 Edens, J. F., Buffington‐Vollum, J. K., Keilen, A., Roskamp, P., & Anthony, C. (2005). Predictions of future dangerousness in capital murder trials: Is it time to ‘disinvent the wheel?’. *Law and Human Behavior, 29*(1), 55–86; Mercado, C. C., Bornstein, B. H., & Schopp, R. F. (2006). Decision‐making about volitional impairment in sexually violent predators. *Law and Human Behavior, 30*(5), 587–602.

43 Hilton, N. Z., & Simmons, J. L. (2001). The influence of actuarial risk assessment in clinical judgments and tribunal decisions about mentally disordered offenders in maximum security. *Law and Human Behavior, 25*(4), 393–408; Monahan, J., & Skeem, J. L. (2016). Risk assessment in criminal sentencing. *Annual Review of Clinical Psychology, 12*(1), 489–513.

44 This is a problem with all attempts to combat risk in these populations, not just neurocorrection. Yet, I embrace the implication that a proportionality requirement might afford insufficient protections against abuse in these other instances too.

45 Stürup‐Toft, S., O'Moore, E., & Plugge, E. H. (2018). Looking behind the bars: Emerging health issues for people in prison. *British Medical Bulletin, 125*(10), 15–23; Stern, V. (2001). Problems in prisons worldwide, with a particular focus on Russia. *Annals of the New York Academy of Sciences, 953b*(1), 113–119.

46 Human Rights Watch. (2015). *Callous and cruel: Use of force against inmates with mental disabilities in US jails*. Human Rights Watch; Wolff, N., & Shi, J. (2009). Contextualization of physical and sexual assault in male prisons: Incidents and their aftermath. *Journal of Correctional Health Care, 15*(1), 58–77.

47 See Douglas, op. cit. note 8, following Eyal, N. (2009). Is the body special? Review of Cécile Fabre, Whose body is it anyway? Justice and the integrity of the person. *Utilitas, 21*(2), 233–245.

48 De Marco & Douglas, op. cit. note 9.

49 I thank an anonymous reviewer for pressing this point.

50 For some examples of this medical and public policy literature see World Health Organization. (2012). *Measurement of and target‐setting for well‐being: An initiative by the WHO Regional Office for Europe, report of the second meeting of the expert group*. World Health Organization Regional Office for Europe; Global Council for Happiness and Wellbeing. (2019). *Global happiness and wellbeing policy report 2019*. https://www.ido-consulting.com/wp-content/uploads/2019/03/GHWPR19.pdf; Department of Health and Social Care UK. (2014). *Wellbeing and why it matters to healthy policy*. Government of the United Kingdom. https://assets.publishing.service.gov.uk/government/uploads/system/uploads/attachment_data/file/277566/Narrative__January_2014_.pdf; Department of Education and Skills Ireland. (2019). *Wellbeing policy statement and framework for practice* 2018–2023. Rialtas na hEireann (Government of Ireland). https://www.education.ie/en/Publications/Policy-Reports/wellbeing-policy-statement-and-framework-for-practice-2018%E2%80%932023.pdf; Ministry of Education New Zealand. (2020). *Wellbeing in education*. https://www.education.govt.nz/our-work/overall-strategies-and-policies/wellbeing-in-education/

51 I acknowledge that were one to accept a pure rehabilitation or moral education theory of punishment, then the expectation of offender benefit would not be problematic as these theories consider the benefitting of offenders to be a legitimate aim of punishment (sometimes even its justifying aim, see Hampton, J. (1984). The moral education theory of punishment. *Philosophy and Public Affairs, 13*(3), 208–238). However, given that these theories are no longer dominant nor popular within legal philosophy, the question at issue here is whether beneficial neurocorrection would prevent the realization of the (commonly defended) retributive, deterrent or denunciatory goals of punishment and I maintain that it would not.

52 See Birks, D. (2021). Paternalism as punishment. *Utilitas, 33*(1), 35–52; and Hanna, N. (2014). Facing the consequences. *Criminal Law and Philosophy, 8*(3), 589–604, for a defence of this claim.

53 Markel, D., & Flanders, C. (2010). Bentham on stilts: The bare relevance of subjectivity to retributive justice. *California Law Review, 98*(3), 907–988, p. 968.

54 I am grateful to an anonymous reviewer for pointing this out.

55 This is the understanding of paternalism advanced by Quong, J. (2010). *Liberalism without perfection*. Oxford University Press, p. 80.

56 See Bullock, E. (2015). A normatively neutral definition of paternalism. *The Philosophical Quarterly, 65*(258), 10–11; Clarke, S. (2002). A definition of paternalism. *Critical Review of International Social and Political Philosophy, 5*(1), 82; Grill, K. (2007). The normative core of paternalism. *Journal of Moral, Legal and Social Philosophy, 13*(4), 442, for this understanding.

